# Folate Receptor Alpha (FRα/FOLR1) in Cancer: Diagnostic, Prognostic or Predictive Biomarker? A Critical Review of Its Clinical Utility

**DOI:** 10.3390/ijms27219720

**Published:** 2026-10-29

**Authors:** Patrycja Kapczuk, Zuzanna Marcinowska, Maciej Kołodziejczak, Donata Szubert

**Affiliations:** Department of Biochemistry and Medical Chemistry, Pomeranian Medical University in Szczecin, Powstańców Wlkp. 72, 70-111 Szczecin, Poland; patrycja.kapczuk@pum.edu.pl (P.K.); zuzanna.marcinowska@pum.edu.pl (Z.M.); maciej.kolodziejczak@pum.edu.pl (M.K.)

**Keywords:** biomarkers, diagnosis, prognosis, prediction

## Abstract

Folate receptor alpha (FRα) plays a key role in folate homeostasis. Folate is involved, among other processes, in nucleotide biosynthesis, and the marked difference between FRα expression in certain cancers and under physiological conditions gives this receptor potential as a cancer biomarker. However, the current state of knowledge points to a specific role for this marker and defines its potential place in cancer diagnosis and treatment. This review aims to critically evaluate the available evidence regarding the clinical utility of FRα. Analysis of the literature indicates that despite differences in expression, the sensitivity and specificity of FRα are insufficient for its use as a standalone diagnostic marker. In contrast, its potential as a prognostic marker is strongly dependent on cancer type. The strongest evidence of clinical utility concerns membranous FRα protein expression assessed by a validated immunohistochemical companion diagnostic assay for selecting patients with platinum-resistant ovarian cancer for mirvetuximab soravtansine treatment. In contrast, the predictive relevance of FRα for other targeted therapeutic strategies remains under investigation. Overall, FRα should be regarded as a clinically actionable biomarker in a defined therapeutic setting and as a promising, but not universally validated, diagnostic, prognostic, and predictive candidate in other oncological applications.

## 1. Introduction

Folate receptor alpha (FRα) has emerged as a molecular target of considerable interest in oncology. Its relatively restricted distribution in many normal adult tissues and elevated expression in several epithelial malignancies have prompted investigations into its diagnostic, prognostic, and predictive significance. However, receptor expression alone does not establish clinical biomarker utility, and the strength of evidence differs considerably across cancer types and applications [1,2,3,4,5].

FRα/FOLR1 can be investigated using several distinct analytical approaches, including the assessment of *FOLR1* mRNA in tissue, immunohistochemical detection of membranous FRα protein, measurement of soluble FRα in biological fluids, and folate receptor-targeted molecular imaging. These modalities reflect different biological properties and cannot be considered interchangeable. In particular, receptor-targeted imaging may not be specific for FRα, and the detection of receptor expression must be distinguished from evidence of diagnostic accuracy or clinical benefit [6,7,8,9,10].

For the purposes of clinical interpretation, biomarkers may serve diagnostic, prognostic, or predictive functions. Diagnostic utility requires evidence that a marker can reliably identify or characterize disease; prognostic utility concerns associations with clinical outcomes independently of treatment; and predictive utility requires evidence that a biomarker identifies patients more likely to benefit from a particular therapy [11,12,13,14,15]. This distinction is especially important for FRα, which is both a potential biomarker and a therapeutic target. Although selected FRα-directed therapeutic strategies, particularly antibody–drug conjugates, have entered clinical practice, the clinical validity of individual FRα measurements remains dependent on the specific assay, cancer setting, and treatment [16].

Therefore, this critical narrative review evaluates the clinical utility of FRα/FOLR1 in cancer diagnosis, prognosis, and the prediction of therapeutic response. We examine differences in biological material and analytical methods, the strength and limitations of available clinical evidence, and the extent to which findings have been validated for clinical decision-making. Our objective is to distinguish established applications from investigational approaches without equating molecular expression or target engagement with demonstrated biomarker utility [1,5,10,17,18].

## 2. Literature Search Strategy

This study was conducted as a critical narrative review aimed at evaluating the clinical utility of folate receptor alpha (FRα/FOLR1) as a diagnostic, prognostic, and predictive biomarker in oncology. The literature search was designed to identify relevant molecular, experimental, and clinical evidence, with particular emphasis on the interpretation, analytical assessment, and clinical validation of FRα-related biomarkers.

Relevant publications were identified through searches of PubMed/MEDLINE, Web of Science, Scopus, and Embase. The initial literature search was conducted in July 2026 and was subsequently updated in October 2026 to incorporate recent and clinically relevant evidence. The search primarily covered publications from 2010 to 2026, with earlier seminal studies included when necessary to provide essential information on receptor structure, ligand binding, tissue distribution, and biological functions. Search terms included “folate receptor alpha”, “FRα”, “FOLR1”, “folate receptor 1”, and “folate-binding protein”, combined with cancer-related terms such as “cancer”, “neoplasm”, “tumor”, and “carcinoma”. Additional search combinations included “diagnosis”, “prognosis”, “survival”, “predictive biomarker”, “treatment response”, “molecular imaging”, “immunohistochemistry”, “soluble folate receptor alpha”, “targeted therapy”, and “antibody–drug conjugate”. Boolean operators (AND, OR) were applied, with search syntax adapted to the requirements of individual databases. Additional relevant publications were identified through reference-list screening.

The literature considered included fundamental molecular studies, in vitro and in vivo experimental investigations, observational studies, diagnostic and imaging studies, clinical trials, and relevant reviews and meta-analyses. Priority was given to original studies directly addressing FRα/FOLR1 expression, its clinical associations, or its potential relevance to patient selection and treatment response. Publications unrelated to FRα/FOLR1 or its oncological applications, duplicate reports, and studies lacking sufficient information for meaningful interpretation were excluded. Studies involving non-malignant conditions were considered only when they provided essential biological or methodological context.

The selected evidence was critically evaluated with particular attention to cancer type, study design, biological material, analytical method, definition of FRα positivity, clinical endpoints, and treatment context. Diagnostic findings were considered in relation to assay performance and clinical applicability; prognostic findings were interpreted with attention to the consistency and independence of reported associations; and predictive findings were evaluated according to their relevance to treatment-specific patient selection and demonstrated clinical benefit. Particular emphasis was placed on distinguishing established clinical applications from preliminary or investigational findings.

Throughout the review, *FOLR1* mRNA expression, membranous FRα protein assessed by immunohistochemistry, circulating soluble FRα (sFRα), and folate receptor-targeted molecular imaging were treated as distinct biomarker modalities. The term FOLR1 refers to the gene or its transcript, whereas FRα denotes the receptor protein. These distinctions were maintained to avoid interpreting biologically and analytically different measurements as interchangeable.

As this work represents a critical narrative review rather than a systematic review or meta-analysis, no formal risk-of-bias assessment or quantitative evidence synthesis was performed. Instead, the available literature was assessed qualitatively to identify the strengths, limitations, and remaining gaps in the clinical validation of FRα/FOLR1 as an oncological biomarker.

## 3. Folate Receptors: Molecular Characteristics and Biological Functions

The human folate receptor family comprises four isoforms: FRα, FRβ, FRγ, and FRδ, encoded by the *FOLR1*, *FOLR2*, *FOLR3*, and *FOLR4* genes, respectively. In the case of folate receptor alpha, the gene symbols *Folr1* and *Folbp1* are also encountered in the literature; however, these designations refer to mouse experimental models rather than humans [19]. FRα and FRβ are anchored to the cell membrane via a glycosylphosphatidylinositol (GPI) moiety, from which they mediate the transport of folate into the cell by endocytosis, followed by ligand release. In contrast, the gamma isoform (FRγ) is a protein secreted into the extracellular space, whereas folate receptor delta (FRδ) remains the least well-characterized member of this family [20].

FRα is a globular glycoprotein with a secondary structure comprising four long α-helices (α1, α2, α3, and α6), two short α-helices (α4 and α5), four short β-strands (β1–β4), and numerous loop regions. The mature receptor has an apparent molecular weight of approximately 38–42 kDa and is strongly stabilized by eight disulfide bonds formed by 16 conserved cysteine residues. The core domain is formed primarily by helices α1, α2, α3, and α5, which are interconnected and stabilized by disulfide bonds. Folic acid is a synthetic, oxidized form of folate, whereas naturally occurring folates are predominantly present as reduced derivatives, including 5-methyltetrahydrofolate (5-MTHF), the major circulating folate form in humans. FRα binds folates at a 1:1 ratio with very high affinity (Kd < 10^−9^ M) and recognizes both folic acid and physiologically relevant reduced folates, including 5-MTHF. Crystallographic analysis of the FRα–folic acid complex revealed a long, deep, and open ligand-binding pocket formed by α-helical, β-strand, and loop elements of the receptor. This pocket exhibits a high degree of spatial complementarity with the ligand. The pteridine ring and para-aminobenzoate moiety of folic acid are tightly anchored within the receptor through an extensive network of hydrogen bonds and π–π interactions, whereas the glutamate moiety remains partially solvent-exposed at the pocket entrance. This structural arrangement permits modification or conjugation through the exposed glutamate moiety while preserving interactions of the pteroate portion with the receptor, providing a structural basis for the development of folate-linked imaging and therapeutic agents. Although the overall architecture of the folate-binding pocket and many ligand-interacting residues are conserved among folate receptor subtypes, FRα and FRβ differ in their binding affinities for physiologically relevant folate species. These differences should therefore be interpreted primarily in terms of ligand affinity and recognition rather than as evidence of fundamentally different ligand-binding pocket architectures [21,22].

Under physiological conditions, folate receptors are highly expressed in the placenta, whereas their expression in adults is restricted and generally occurs at relatively low levels. These receptors have been detected, among other sites, in the proximal renal tubules, urinary bladder, central nervous system, male and female reproductive organs, salivary glands, colon, lungs, basolateral membranes of retinal pigment epithelial cells, mesothelium, and tissues of the lymphatic and hematopoietic systems [4,6].

**Figure 1:**
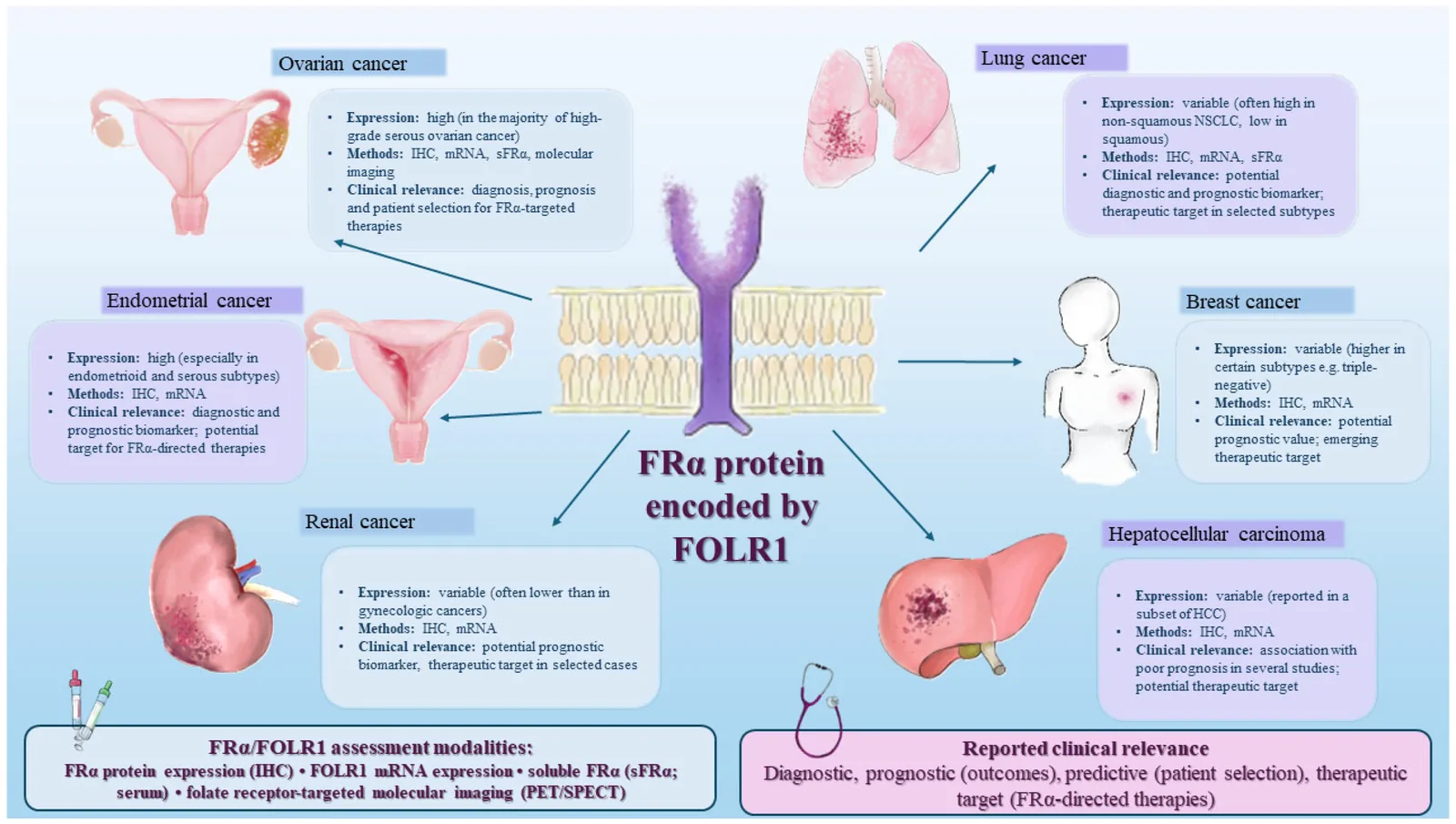
FRα/FOLR1 expression and its reported clinical relevance across selected cancer types. The figure summarizes the principal approaches used to assess FRα/FOLR1 in selected malignancies discussed in this review, including membranous FRα protein expression assessed by immunohistochemistry (IHC), FOLR1 mRNA expression, circulating soluble FRα (sFRα), and folate receptor-targeted molecular imaging. Diagnostic, prognostic, predictive, and therapeutic relevance is presented according to the respective biomarker modality to emphasize that these biologically and analytically distinct measurements should not be interpreted interchangeably. The figure was created by the authors using Sketchbook (version 6.2.6) and FireAlpaca (version 2.16.3).

The *FOLR1* gene encodes FRα, which has a restricted tissue expression profile. Its expression is found predominantly in polarized epithelial cells, where the receptor is localized to the apical domain of the cell membrane. This distribution enables the selective uptake of folates from the extracellular space and is observed, among other tissues, in the proximal renal tubular epithelium, pulmonary epithelial cells, choroid plexus, reproductive tissues, secretory glands, and placental trophoblasts [23]. High expression of three folate receptor isoforms (α, β, and γ) is also observed in cancer cells. Overexpression of FRα has been demonstrated in ovarian, cervical, metastatic endometrial, brain, pancreatic, breast, lung, renal, colorectal, and bladder cancers, as well as lymphoma. FRβ overexpression has been reported in acute myeloid leukemia (AML), chronic myeloid leukemia (CML), ovarian, uterine, breast, renal, liver, skin, and gastric cancers, glioma, astrocytoma, and metastatic pancreatic cancer. FRγ has been detected in hematological malignancies and epithelial tumors of the ovary, endometrium, and cervix and may be secreted into the bloodstream in very small amounts, making its detection difficult [10,24]. Importantly, the level of FRα/FOLR1 expression and its reported clinical significance vary across cancer types and depend on the biomarker modality and analytical method used. An overview of FRα/FOLR1 assessment and its reported clinical relevance in selected malignancies is presented in Figure 1. Although increased FRα expression has been documented in multiple malignancies, the biological and clinical interpretation of these findings requires consideration of the cellular compartment and analytical method used. Elevated FOLR1 mRNA levels do not necessarily correspond to high membranous FRα protein abundance, while tissue-level measurements may be influenced by tumor heterogeneity and the presence of non-malignant cells. Furthermore, the expression of other folate receptor isoforms, particularly FRβ in activated macrophages, may complicate the interpretation of folate receptor-targeted imaging and ligand-binding studies. Consequently, receptor expression should be characterized using methods appropriate to the intended clinical application rather than interpreted as a single, uniform biomarker measurement [7,8,23,24,25].

Cancer cells are also capable of releasing FRα, which occurs in the circulation in a soluble form. O’Shannessy et al. [26] developed electrochemiluminescent assays for the detection of soluble FRα and evaluated its potential diagnostic utility in epithelial ovarian cancer. Their findings suggested that serum FRα may provide complementary diagnostic information, particularly when combined with other circulating biomarkers, although further clinical validation is required [26]. Methods for detecting the receptor are not limited to antibody-based immunological techniques. Probes are currently being developed to enable rapid, selective, and stable labeling of endogenous FRα without disrupting the function of living cells. Fang et al. [27] designed a protein-labeling probe for FRα that binds to the receptor and subsequently covalently labels it with a fluorophore. This approach enables long-term monitoring of the fate of the same protein using fluorescence microscopy and electrophoresis [27]. Furthermore, O’Shannessy et al. [7] demonstrated that newly developed monoclonal antibodies against FRα enable a more sensitive and reliable assessment of its expression in tumor tissues. The binding properties of these antibodies were shown to depend on receptor conformation and the accessibility of its antigenic determinants, which are influenced, among other factors, by post-translational modifications, including glycosylation and GPI anchoring. These findings support the importance of FRα as a biomarker and highlight the need to use validated diagnostic tools when assessing its expression [7].

The primary role of FRα is folate uptake through endocytosis. It also contributes to efficient folate uptake, particularly under conditions of low extracellular folate concentrations or increased cellular proliferation, when the demand for folates rises because of their role as cofactors in one-carbon metabolism, which is essential for methylation reactions and nucleotide and DNA biosynthesis. In the choroid plexus, FRα mediates folate transport from the basolateral domain to the apical surface of epithelial cells, from which receptor–folate complexes may be transported within exosomes across the blood–brain barrier, thereby ensuring folate availability to the central nervous system. In the placenta, the receptor participates in the selective transport of folates from the maternal circulation to the fetus, supporting normal embryonic development. In the kidneys, FRα contributes to folate reabsorption from the primary urine in the renal tubules, limiting urinary folate loss and helping maintain systemic folate homeostasis [2,23].

Nawaz and Kipreos [23] highlighted the multifunctional nature of FRα, which may participate in non-canonical signaling pathways regulating cell proliferation, survival, and differentiation, including JAK–STAT3, ERK1/2, transcriptional regulation associated with nuclear translocation of FRα, Wnt, TGF-β, NOTCH3, FGFR1, LYN, and Gαi-3. Following ligand binding, the receptor may participate in the formation of protein complexes that initiate phosphorylation cascades, including through interaction with the gp130/JAK complex leading to STAT3 activation as well as through modulation of the PGR/SRC/ERK1/2/NF-κB/p53 axis involved in the regulation of proliferation and cellular responses. Other pathways, including Wnt, TGF-β, NOTCH3, FGFR1, LYN, and Gαi-3, have been proposed on the basis of experimental observations; however, their direct dependence on FRα remains insufficiently documented [23].

FRα has also been attributed a role as a transcription factor. Boshnjaku et al. [28] demonstrated that the receptor can undergo internalization and translocation to the cell nucleus, where it binds to specific DNA sequences within gene promoter regions. Receptor-binding sites within cis-regulatory elements were identified in the promoter regions of Fgfr4 and Hes1. These findings may contribute to a new understanding of the role of FRα beyond that traditionally attributed to the receptor. The authors suggest that it may be involved not only in embryonic development, for example, in the nervous system, but also in tumorigenesis, in which FRα is frequently overexpressed and may represent a potential therapeutic target [23,28].

Taken together, these findings indicate that FRα may participate in biological processes extending beyond folate transport, including intracellular signaling and transcriptional regulation. Nevertheless, the mechanistic evidence supporting these functions is derived predominantly from experimental models, and the extent to which individual pathways contribute to tumor behavior may vary according to cellular context. Therefore, the biological involvement of FRα in proliferation, survival, or signaling should not be interpreted as direct evidence of its clinical utility as a diagnostic, prognostic, or predictive biomarker. Establishing such utility requires studies linking clearly defined FRα/FOLR1 measurements to clinically relevant outcomes in appropriately characterized patient populations [23,28].

## 4. FRα/FOLR1 as a Diagnostic Biomarker

Folate receptor alpha has been investigated as a potential diagnostic marker; however, its clinical utility requires further validation in diverse patient populations. Available studies have demonstrated the potential use of folate-receptor-targeted tracers as imaging biomarkers [8,29,30,31]. Importantly, evaluation of this evidence requires a clear distinction between biologically and analytically different biomarker modalities.

FOLR1 mRNA expression, membranous FRα protein assessed by IHC, soluble FRα (sFRα), and folate-receptor-targeted molecular imaging represent distinct measurements and should not be interpreted interchangeably. *FOLR1* mRNA reflects transcriptional activity, whereas membranous FRα provides information on receptor abundance and localization. In contrast, sFRα represents a circulating analyte, whereas molecular imaging provides spatial information on receptor-accessible lesions. These modalities therefore require separate interpretation according to the analytical method and intended clinical application. Representative examples of each of these biomarker modalities and their potential clinical relevance are discussed in the following sections.

### 4.1. Folate Receptor-Targeted Molecular Imaging

One distinct diagnostic application of folate receptor targeting is molecular imaging. Vlahov et al. (2012) described FRα as a biomarker associated with patient selection for targeted therapies and potentially as an imaging and prognostic marker, rather than as a conventional diagnostic marker indicating the presence or absence of disease [32]. They also emphasized its role in diagnostic imaging for tumor localization, assessment of heterogeneity in receptor expression, and the identification of lesions with the greatest dependence on folate receptor expression [32,33]. In nuclear medicine imaging, folate receptor expression can be exploited for the localization of neoplastic lesions using radiolabeled folic acid analogues or 5-methyltetrahydrofolate (5-MTHF) (Table 1).

Most folic acid-based radiotracers target folate receptors without intrinsic selectivity for FRα over FRβ. Consequently, tracer uptake may reflect not only FRα-positive tumor cells but also FRβ-expressing activated macrophages in inflammatory or tumor-associated tissues. Among the tracers listed, ^18^F-6R-aza-5-MTHF has been specifically evaluated for FRα versus FRβ binding and demonstrated preferential FRα selectivity [25]. Therefore, uptake of non-subtype-selective folate radiotracers should not be interpreted as a direct quantitative measure of tumor-cell FRα expression.

Lu et al. [41] also demonstrated that ^99m^Tc-labeled folate conjugates have high affinity for FR-positive tumor cells in vitro and exhibit specific receptor-dependent binding that can be inhibited by free folic acid. In an animal model, high tracer accumulation was observed in FR-positive tumors, whereas accumulation in FR-negative and healthy tissues was low, resulting in a favorable tumor-to-background ratio. These findings supported the potential utility of folate receptor-targeted tracers for scintigraphic tumor visualization [41]. Ocak et al. [42] investigated the combination of different imaging techniques, such as optical and nuclear imaging, for multimodal folate receptor-targeted imaging of ovarian cancer in a novel syngeneic mouse model. Their findings supported the potential value of multimodal imaging approaches for tumor detection and differentiation from surrounding tissues [42]. Chen et al. [40], in turn, emphasized that the folate-NOTA-Al^18^F complex enables stable labeling of folate with fluorine-18, which represents an important advantage over other radioisotope-labeling methods. The developed radiotracer showed potential for the non-invasive imaging of FR-positive tumors, and potentially for identifying patients who may be suitable for folate receptor-targeted therapies [40]. Radiofolates were initially developed and investigated for the molecular imaging of rheumatoid arthritis, osteoarthritis, and atherosclerotic lesions, where they enable the detection of activated macrophages overexpressing FRβ in affected tissues. More recently, they have also been investigated for targeting cancer cells characterized by increased FRα expression. Several radiotracers have been developed for folate receptor-targeted nuclear medicine imaging. The three most frequently described agents that have reached clinical investigation are ^111^In-DTPA-folate, ^99m^Tc-EC20 (etarfolatide), and ^18^F-AzaFol (3′-aza-2′-[^18^F]fluorofolic acid). Among the tracers listed in Table 1, ^18^F-AzaFol is of particular interest because PET provides greater sensitivity and spatial resolution than SPECT and enables the quantitative assessment of radiotracer uptake in folate receptor-accessible lesions. However, because ^18^F-AzaFol is not intrinsically selective for FRα over FRβ, its uptake should not be interpreted as a direct quantitative measure of tumor-cell FRα expression [25,36]. In contrast, ^99m^Tc-EC20 (etarfolatide) remains the most extensively clinically investigated SPECT radiopharmaceutical for folate receptor imaging, whereas ^111^In-DTPA-folate played an important role as the first folate ligand used clinically, although it is now primarily of historical and research significance [34,35,36,43].

Despite the promising results obtained with folate receptor-targeted imaging, several limitations restrict its interpretation as a cancer-specific diagnostic approach. First, most folate-based radiotracers are not exclusively selective for FRα and may also accumulate in tissues containing FRβ-expressing activated macrophages. Second, radiotracer uptake reflects not only receptor expression but also tracer delivery, tissue perfusion, receptor accessibility, and pharmacokinetic properties. Third, many published investigations involve preclinical models or relatively small clinical cohorts, limiting the generalizability of diagnostic performance estimates. Consequently, the visualization of folate receptor-positive lesions should not be considered equivalent to histopathological confirmation of malignancy or to quantitative determination of membranous FRα expression [8,25,30,31,33,36,44].

The clinical value of folate receptor-targeted imaging may therefore lie primarily in complementary lesion characterization, assessment of receptor-accessible disease, and potential support for treatment planning rather than in replacing established diagnostic procedures. Prospective studies comparing these imaging approaches with validated reference standards are needed to establish their incremental diagnostic value, reproducibility, and suitability for specific clinical indications [8,25,31,33].

### 4.2. Tissue-Based and Circulating FRα/FOLR1 Biomarkers

Beyond molecular imaging, the diagnostic relevance of FRα/FOLR1 has also been investigated using tissue-based and circulating biomarkers. From a clinical perspective, the value of a diagnostic marker depends not only on its association with the presence of disease, but also on the analytical method used, the accessibility of the biological material, and its diagnostic sensitivity and specificity. Numerous studies have therefore evaluated FRα/FOLR1 in tumor tissue as well as soluble FRα in serum.

Ahmed et al. [45] assessed serum thymidine kinase 1 (TK1) and FRα as potential circulating biomarkers of prostate cancer. Both parameters were significantly elevated in patients with prostate cancer compared with controls; however, their diagnostic performance differed markedly. TK1 demonstrated very high diagnostic performance and correlated with disease stage and tumor aggressiveness, supporting its potential as both a diagnostic and prognostic marker. FRα, in contrast, showed only moderate diagnostic accuracy, limited sensitivity and specificity, and no significant correlation with clinicopathological parameters such as disease stage or tumor aggressiveness. These findings suggest that serum FRα may serve only as an adjunctive biomarker rather than as a standalone diagnostic test for prostate cancer. The authors emphasized that the most promising results were obtained by combining TK1 with PSA, which may improve the accuracy of prostate cancer diagnosis [45]. Kurosaki et al. [9] evaluated soluble FRα (sFRα) as a circulating biomarker in epithelial ovarian cancer (EOC) and reported significantly higher serum sFRα concentrations in patients with EOC than in healthy women and patients with benign ovarian lesions. A positive correlation was also observed between serum sFRα concentrations and FRα expression in tumor tissue, suggesting that soluble FRα reflects biological characteristics of the tumor. At the same time, the diagnostic sensitivity of sFRα was insufficient for it to replace CA-125 or serve as a standalone diagnostic tool. These findings suggest that sFRα may have the greatest clinical value as a complementary circulating biomarker rather than as a standalone diagnostic marker [9]. In contrast to circulating sFRα, tissue-based assessment of membranous FRα by immunohistochemistry (IHC) represents a distinct biomarker modality with direct relevance to patient selection for FRα-targeted therapy. Scheiter et al. [10] conducted the first interlaboratory quality assessment of immunohistochemical (IHC) FRα testing and demonstrated substantial differences in results obtained using different antibodies and staining protocols. Assessment of membranous FRα expression is of particular clinical relevance because the result may determine whether patients with ovarian cancer are eligible for FRα-targeted therapy. In the United States, the validated VENTANA FOLR1 (FOLR1-2.1) RxDx assay allows the identification of tumors in which at least 75% of cells exhibit moderate-to-strong membranous FRα expression, which constitutes a criterion for treatment with mirvetuximab soravtansine. However, the study showed that differences in antibody sensitivity, interpretation of staining intensity, and diagnostic experience may result in incorrect classification of FRα status. Consequently, reliable use of FRα as a biomarker requires validated IHC methods, appropriate quality-control procedures, and a standardized interpretation of results. The study therefore emphasizes that the clinical utility of membranous FRα as a tissue-based biomarker depends not only on its biological expression, but also on the analytical reliability and standardization of the IHC method used [10].

In hepatocellular carcinoma (HCC), FRα has been investigated using both circulating and tissue-based approaches. In a cohort study evaluating its diagnostic and prognostic relevance, serum FRα showed diagnostic performance for HCC comparable to that of alpha-fetoprotein (AFP), while combining the two markers improved diagnostic accuracy. In the same study, higher FRα expression was also observed in tumor tissue than in non-neoplastic tissue and was associated with aggressive subtypes and activation of the p53, DNA repair, Myc, E2F, and PI3K/AKT/mTOR pathways [46].

Taken together, the available evidence indicates that the diagnostic relevance of FRα/FOLR1 depends strongly on the cancer type, biological material, analytical method, and intended clinical application. Although increased circulating FRα concentrations or tissue FOLR1/FRα expression have been reported in several malignancies, statistically significant differences between patients and controls do not necessarily translate into clinically meaningful diagnostic performance. In particular, the available studies do not establish soluble FRα as a reliable standalone cancer detection marker or demonstrate consistent incremental benefit over established diagnostic approaches. Tissue-based assessment of membranous FRα serves a fundamentally different purpose, particularly in identifying patients eligible for FRα-targeted treatment, whereas folate receptor-targeted imaging reflects tracer-accessible receptor expression and may be influenced by FRβ-positive inflammatory cells. These modalities should therefore not be considered interchangeable. Future studies should prioritize analytical standardization, externally validated diagnostic thresholds, appropriate control populations, and direct comparisons with existing clinical pathways to determine whether FRα/FOLR1 assessment provides additional information capable of improving patient management [9,10,45].

## 5. FRα/FOLR1 as a Predictive Biomarker and Therapeutic Target

From a therapeutic perspective, the differential expression of FRα between malignant and non-malignant tissues provides a biological rationale for the development of receptor-targeted anticancer strategies. Elevated FRα expression in several epithelial malignancies may facilitate the selective recognition of tumor cells and the delivery of therapeutic agents. Nevertheless, FRα is not exclusively expressed in malignant tissues, and receptor expression alone does not guarantee tumor-specific drug accumulation, therapeutic efficacy, or the absence of off-target toxicity. Therefore, the clinical implementation of FRα-targeted therapies requires the careful consideration of receptor distribution, expression heterogeneity, and treatment-specific mechanisms of action [5,47,48].

Importantly, the presence of FRα as a therapeutic target should not automatically be interpreted as evidence of its predictive value. A clinically useful predictive biomarker must be supported by evidence demonstrating that its assessment informs treatment selection and identifies patients likely to benefit from a particular intervention. Currently, the strongest clinical evidence concerns membranous FRα protein expression assessed using a validated immunohistochemical companion diagnostic assay for patient selection for mirvetuximab soravtansine. In contrast, the predictive relevance of FRα expression for newer antibody–drug conjugates, CAR-T cells, radionuclide therapies, and other receptor-directed approaches remains under investigation [10,17,18,49,50,51,52,53,54,55,56].

Potential FRα-based therapeutic strategies include:

- blocking FRα, thereby limiting FRα-mediated folate uptake and/or receptor-dependent signaling;
- using the receptor as a route for delivering therapeutic agents to tumor cells, including appropriate analogues or conjugates linked to folic acid or monoclonal antibodies;
- using CAR-T technology, involving modification of a patient’s T lymphocytes to recognize and eliminate cells expressing FRα.

Importantly, FRα represents only one of several cellular folate uptake pathways. Reduced folates can also be transported by the reduced folate carrier (RFC/SLC19A1) and the proton-coupled folate transporter (PCFT/SLC46A1). Therefore, blockade of FRα should not be interpreted as complete inhibition of cellular folate uptake or as necessarily resulting in folate deprivation, as alternative transport pathways may maintain intracellular folate availability. This concept is supported by experimental studies in epithelial ovarian cancer models, in which FRα knockdown markedly reduced FRα-mediated folate binding and uptake, while substantial PCFT-mediated transport was retained. Thus, the biological consequences of FRα blockade are likely to depend, at least in part, on the relative contribution of alternative folate transport systems [57,58].

A further argument supporting the use of FRα as a therapeutic target is that its expression appears to remain at similar levels in primary tumors and in recurrent and metastatic lesions [48,49].

### 5.1. Folate-Targeted Drug Delivery and Radionuclide Therapy

One of the most intensively investigated strategies involves folate–drug conjugates (FDCs), in which folic acid acts as a targeting ligand that enables specific recognition of FRα-positive cells and delivery of the drug into the tumor cell. Folate–drug conjugates such as vintafolide are small-molecule ligand–drug conjugates in which folate serves as the receptor-targeting moiety and should therefore be distinguished from antibody–drug conjugates (ADCs), in which target recognition is mediated by a monoclonal antibody. These conjugates consist of three components: a ligand, a linker, and a cytotoxic payload [59,60,61].

An example of this approach is vintafolide (EC145), a conjugate of a folic acid analogue with desacetyl vinblastine monohydrazine (DAVLBH), whose activity is based on FRα-dependent internalization followed by intracellular release of the active cytotoxic compound. The molecule consists of a folate ligand linked to the cytotoxic compound through a peptide linker and a reducible disulfide-containing self-immolative linker system. Following endocytic internalization of the complex by an FRα-positive cell, the active DAVLBH metabolite is released, disrupting mitotic spindle formation and thereby inhibiting proliferation and inducing tumor cell death. Preclinical studies demonstrated that the anticancer activity of vintafolide is strongly dependent on the presence of FRα, whereas cells lacking receptor expression show limited sensitivity to the conjugate. An excess of free folic acid blocked the binding and cytotoxic effects of vintafolide, confirming the specificity of its ligand–receptor-dependent mechanism [61].

However, vintafolide has not received clinical approval for the treatment of cancer, including platinum-resistant ovarian cancer. Its development was halted during late-stage clinical investigation (phase III) because of insufficient efficacy in a confirmatory trial [48].

FRα imaging provided a basis for the development of targeted radionuclide therapy (TRT). In TRT, rather than radionuclides emitting γ radiation or positrons, isotopes emitting β^−^ or α particles are used to induce irreversible DNA damage in tumor cells. FRα-targeted radiopharmaceuticals may therefore represent another therapeutic strategy, in which a folate ligand is conjugated to a radionuclide emitting cytotoxic radiation. Commonly used tracers include ^99m^Tc-etarfolatide, ^18^F-AzaFol, and other radiolabeled folate analogues, while radionuclides emitting β or α radiation, such as ^177^Lu and ^161^Tb, are currently being investigated for therapeutic applications [31].

Although FRα represents an attractive target for radionuclide therapy because of the high affinity of folate ligands and the receptor’s capacity for internalization, FRα-targeted radiopharmaceuticals remain at the clinical and preclinical research stages. It should also be noted that tumor cells do not all express FRα at the same level. Consequently, pretreatment PET/SPECT imaging is currently considered particularly important. A major limitation is physiological FRα expression in the kidneys, which results in increased renal uptake of radiofolates and creates a risk of nephrotoxicity. The development of new ligands, chelators, and strategies for reducing renal accumulation may enable the future use of FRα as a target for radionuclide therapy [31].

This approach allows radioactive energy to be delivered selectively to receptor-expressing cells, resulting in DNA damage and the activation of pathways leading to tumor cell death. A similar concept is being developed within theranostic approaches, in which analogous molecules may be used both to image FRα expression and subsequently to deliver targeted therapy.

From a biomarker perspective, the clinical interpretation of folate receptor-targeted radionuclide therapy requires particular caution. Although radiolabeled folate derivatives can demonstrate receptor-associated uptake in tumors, tracer accumulation does not necessarily correspond to membranous FRα expression measured by immunohistochemistry or predict therapeutic response. Imaging signals may be influenced by ligand affinity, receptor accessibility, tissue perfusion, tracer pharmacokinetics, and uptake by non-malignant cells, including FRβ-positive activated macrophages. Furthermore, the relationship between pretreatment tracer uptake, absorbed radiation dose, and subsequent tumor response has not been sufficiently validated for routine treatment selection [8,24,25,30,31,33].

A major translational challenge is the accumulation of folate-based radiopharmaceuticals in the kidneys, which may restrict the therapeutic window. Consequently, successful receptor targeting must be considered alongside tumor-to-organ dose ratios and the potential for radiation-induced toxicity. Although theranostic approaches offer opportunities to combine molecular imaging with individualized radionuclide delivery, the clinical utility of folate receptor-targeted imaging as a predictive biomarker for radionuclide therapy remains investigational. Prospective studies integrating quantitative imaging, dosimetry, toxicity assessment, and treatment outcomes are needed before imaging-derived thresholds can be used to guide therapeutic decisions [8,25,30,31,36].

### 5.2. Monoclonal Antibodies and Antibody–Drug Conjugates

FRα is also a target for monoclonal antibodies and antibody–drug conjugates (ADCs). Antibodies directed against the extracellular domain of the receptor enable selective recognition of FRα-positive cells and the delivery of conjugated cytotoxic agents. The mechanism of action of these constructs involves internalization of the receptor–antibody complex, release of the active compound, and induction of effects resulting in inhibition of proliferation or tumor cell death. The best-characterized example, which is also used in the treatment of adult patients with FRα-positive, platinum-resistant epithelial ovarian, fallopian tube, or primary peritoneal cancer, is mirvetuximab soravtansine (MIRV, IMGN853, Elahere^®^). MIRV consists of an anti-FRα IgG1 monoclonal antibody (M9346A) conjugated to the maytansinoid DM4 (ravtansine), a potent microtubule inhibitor, through the disulfide-containing sulfo-SPDB linker. Following binding to FRα on the tumor-cell surface, the FRα–MIRV complex undergoes receptor-mediated internalization and vesicular trafficking to lysosomes. Lysosomal degradation of the antibody is followed by linker-dependent intracellular processing and the release of thiol-containing DM4, which may subsequently undergo methylation to form S-methyl-DM4. Thus, sulfo-SPDB should be distinguished from conventional protease-cleavable peptide linkers used in several newer ADCs, because payload liberation does not rely on direct proteolytic cleavage of a peptide linker. Both DM4 and S-methyl-DM4 disrupt microtubule dynamics, leading to cell-cycle arrest and apoptosis. Moreover, their membrane permeability, particularly that of S-methyl-DM4, contributes to a bystander effect that may extend cytotoxic activity to neighboring tumor cells with lower or heterogeneous FRα expression [17,48].

The clinical development of mirvetuximab soravtansine provides the strongest evidence supporting the clinical utility of FRα assessment for treatment selection. Patient eligibility is based on membranous FRα protein expression determined using the VENTANA FOLR1 (FOLR1-2.1) RxDx immunohistochemical assay, with FRα-high expression defined as moderate-to-strong (2+/3+) membranous staining in at least 75% of viable tumor cells. This assay-specific threshold should not be regarded as universally applicable to other FRα-directed therapeutic agents [10,17].

The phase II SORAYA trial evaluated mirvetuximab soravtansine in patients with FRα-high, platinum-resistant ovarian cancer previously treated with bevacizumab and demonstrated an objective response rate of approximately 32%. Although these results supported the antitumor activity of mirvetuximab soravtansine in a biomarker-selected population, the single-arm design did not permit a direct comparison with alternative therapies or establish whether FRα expression modified the relative treatment effect [17,49].

More definitive comparative evidence was provided by the randomized phase III MIRASOL trial, in which mirvetuximab soravtansine was compared with the investigator’s choice of chemotherapy in patients with FRα-high, platinum-resistant ovarian cancer. Mirvetuximab soravtansine improved the objective response rate (approximately 42% versus 16%), median progression-free survival (5.6 versus 4.0 months), and median overall survival (16.5 versus 12.8 months). These findings demonstrate clinically meaningful benefit in the population selected using the established FRα immunohistochemical criterion [18].

Nevertheless, the distinction between clinical utility for treatment selection and formal biomarker–treatment interaction is important. Because the pivotal studies enrolled patients meeting the FRα-high eligibility criterion, they do not establish the magnitude of relative treatment benefit across the entire spectrum of FRα expression. Accordingly, the validated threshold supports patient selection for mirvetuximab soravtansine but should not be extrapolated to other FRα-targeted agents without independent analytical and clinical validation [10,17,18,49].

Several additional FRα-targeted ADCs have entered clinical development, with programs currently spanning early-phase studies through phase III trials, while some earlier candidates have been discontinued (Table 2).

The clinical development of FRα-directed therapies illustrates that the identification of a molecular target does not necessarily translate into therapeutic success. The discontinuation of vintafolide following insufficient efficacy in late-stage clinical development demonstrates the limitations of assuming that receptor expression alone can reliably predict treatment benefit. Conversely, the successful clinical development of mirvetuximab soravtansine demonstrates that FRα assessment can support an effective biomarker-guided treatment strategy when an appropriate therapeutic construct, analytically validated assay, and clinically evaluated expression threshold are used [17,18,48,49,61].

More recently developed FRα-targeted ADCs, including torvutatug samrotecan (AZD5335), sofetabart mipitecan (LY4170156), and IMGN151, incorporate distinct antibody architectures, linker technologies, and cytotoxic payloads designed to address limitations associated with earlier therapeutic approaches. AZD5335 and LY4170156 employ topoisomerase I inhibitor-based payload strategies, whereas IMGN151 incorporates a biparatopic antibody design and the maytansinoid DM21. These differences may influence receptor binding, internalization, intracellular payload delivery, and antitumor activity in tumors with heterogeneous or relatively low FRα expression [50,51,52,53,54].

Importantly, these agents should not be regarded as a homogeneous therapeutic class with interchangeable biomarker requirements. Their mechanisms of action and emerging clinical development programs suggest that the relationship between FRα expression and therapeutic activity may differ between individual ADCs. Although preclinical studies and ongoing clinical investigations support the evaluation of treatment across different receptor-expression categories, these findings do not establish a universally applicable FRα threshold or demonstrate that expression level is a validated predictor of relative treatment benefit for each agent [50,51,52,53,54].

Consequently, future studies should evaluate the analytical performance of the assays used for patient selection, investigate treatment outcomes across predefined FRα-expression strata, and determine whether receptor expression modifies the magnitude of benefit compared with appropriate control treatments. Such analyses will be essential to establish whether FRα can serve as a treatment-specific predictive biomarker for newer ADCs rather than solely as a molecular target supporting their development [50,51,52,53,54].

### 5.3. Emerging Therapeutic Strategies

Another emerging therapeutic strategy involves genetically engineered T lymphocytes expressing chimeric antigen receptors (CAR-T cells) directed against FRα. Unlike antibody–drug conjugates, which exploit receptor-mediated internalization to deliver cytotoxic payloads, CAR-T cells recognize FRα as a cell-surface antigen and initiate T-cell-mediated cytotoxicity following antigen engagement. This approach provides a biological rationale for targeting FRα-expressing malignancies, particularly ovarian cancer, in which receptor expression is frequently elevated relative to many normal tissues [55,56].

However, the expression of FRα as a target antigen should not be equated with its validated predictive utility. Although receptor expression is necessary for antigen-dependent recognition, the therapeutic efficacy of CAR-T cells depends on multiple additional factors, including antigen density, accessibility and heterogeneity, T-cell trafficking and persistence, and the immunosuppressive properties of the tumor microenvironment. Consequently, the detection of FRα-positive tumor cells does not necessarily indicate that a patient will achieve a clinically meaningful response to FRα-directed cellular therapy [55,56].

Yang et al. (2025) highlighted several challenges associated with the application of CAR-T therapies in solid tumors, including heterogeneous antigen expression, insufficient infiltration of engineered T cells into tumor tissue, and immunosuppressive mechanisms that impair T-cell activity [53]. These limitations are particularly relevant to FRα-directed approaches because intratumoral variation in receptor expression may facilitate the survival and expansion of antigen-low or antigen-negative tumor-cell populations. Furthermore, the persistence of FRα expression during disease progression and following previous therapies requires consideration when evaluating the suitability of this antigen for repeated or sequential treatment [55].

Additional experimental evidence was provided by Choe et al. [56], who investigated FRα-targeted CAR-T cells in osteosarcoma models. The FH FOLR1-CART product demonstrated antigen-dependent cytotoxic activity in vitro and antitumor effects in xenograft models, including patient-derived models. Importantly, the absence of significant cytotoxicity in a FOLR1-negative model supported the specificity of the therapeutic approach. Nevertheless, these findings establish proof-of-concept rather than clinical predictive validity. The observed association between FOLR1 expression and CAR-T activity in experimental systems cannot be directly translated into a clinically applicable expression threshold or used to predict treatment outcomes in patients [56].

A further methodological consideration concerns the distinction between FOLR1 mRNA expression and membranous FRα protein expression. Although gene-expression analyses may support the identification of tumors potentially expressing the target antigen, CAR-T recognition depends on the presence and accessibility of FRα protein at the cell surface. Therefore, transcript abundance alone may not adequately reflect the density, distribution, or functional accessibility of the target antigen. Future clinical development should incorporate standardized assessment of membranous FRα expression, ideally together with the spatial evaluation of intratumoral heterogeneity [55,56].

Overall, FRα-targeted CAR-T therapy represents a promising experimental application of the receptor as an immunotherapeutic antigen. However, the available evidence supports its biological suitability as a therapeutic target rather than its established clinical role as a predictive biomarker. Prospective clinical studies are required to determine whether FRα expression levels, antigen distribution, or other tumor-associated characteristics can reliably identify patients most likely to benefit from this approach [55,56].

The use of FRα also extends to the development of nanotechnology-based drug-delivery systems, including folate-functionalized nanoparticles and nanoemulsions. Such systems may exploit folate receptor-mediated targeting to facilitate the selective delivery of therapeutic compounds to FRα-expressing cells [47,67].

Patel et al. developed nanoemulsions containing lipophilic platinum derivatives whose surfaces were modified with the folate ligand FA–PEG3400–DSPE, enabling selective recognition of FRα and receptor-mediated endocytic internalization of the nanoparticles. In vitro studies showed that the nanoemulsions exhibited cytotoxic activity against both platinum-sensitive cells and models of resistance to platinum compounds, with the NMI-350 formulation containing dimyrisplatin showing the most promising properties. In addition, the incorporation of lapidated Gd-DTPA allowed therapeutic activity to be combined with imaging potential, making the developed system an example of an FRα-targeted theranostic platform. This study therefore supports the use of FRα not only as a marker expressed on the surface of tumor cells, but also as a molecular address that enables the selective delivery of drugs and imaging agents to the tumor [47,67].

Despite these encouraging findings, the results obtained with folate-functionalized nanoemulsions should be interpreted primarily as preclinical evidence supporting receptor-mediated drug delivery rather than as validation of FRα as a predictive biomarker. Enhanced cellular uptake or cytotoxicity in experimental models does not necessarily translate into improved tumor accumulation, therapeutic efficacy, or an acceptable safety profile in patients. The biological behavior of nanocarriers may additionally be influenced by particle size, surface characteristics, systemic clearance, and interactions with non-malignant tissues. Moreover, folate-based targeting is not intrinsically specific to FRα, as other folate receptor isoforms may contribute to ligand binding in certain cellular populations [24,29,47,67].

Therefore, although the study by Patel et al. supports the feasibility of exploiting folate receptor-mediated internalization for therapeutic delivery, it does not establish a clinically applicable FRα-expression threshold for predicting treatment response. Further investigations incorporating quantitative receptor characterization, pharmacokinetic assessment, biodistribution, and in vivo therapeutic efficacy are required before FRα-targeted nanocarriers can be considered a clinically validated biomarker-guided treatment strategy [30,47,67].

Growing preclinical evidence indicates that FOLR1 may contribute to molecular mechanisms underlying sorafenib resistance in hepatocellular carcinoma (HCC), suggesting its potential as a candidate biomarker of treatment response. However, these findings remain mechanistic and preclinical and do not establish FOLR1/FRα as a clinically validated predictor of response to sorafenib. Chu et al. demonstrated that FRα overexpression induces autophagy, a process that enables cancer cells to adapt to treatment-induced stress. Increased autophagy promotes HCC cell survival and attenuates the cytotoxic effects of sorafenib, whereas *FOLR1* knockdown inhibits autophagic flux and restores cellular sensitivity to treatment [68].

The role of *FOLR1* in the acquisition of drug resistance was further supported by Zhao et al., who demonstrated that receptor expression is modulated by the tumor microenvironment. Cancer-associated fibroblasts (CAFs) secrete the chemokine CXCL12, which increases *FOLR1* expression in HCC cells through activation of the CXCL12/CXCR4 axis. Activation of this pathway enhances proliferation, migration, and invasion while reducing sensitivity to sorafenib. Pharmacological inhibition of CXCL12/CXCR4 signaling or suppression of *FOLR1* expression limited the development of the resistant phenotype, indicating that *FOLR1* acts as an effector of signals originating from the tumor microenvironment [69].

Taken together, these experimental findings suggest that FOLR1 may participate in adaptive mechanisms contributing to sorafenib resistance in hepatocellular carcinoma, including autophagy-associated survival pathways and tumor microenvironment-mediated signaling. However, the available evidence does not establish that FOLR1 expression independently predicts clinical response to sorafenib or improves treatment selection in patients. Prospective clinical investigations incorporating standardized FOLR1/FRα assessment and treatment-specific outcomes are therefore required before this proposed association can be translated into a validated predictive biomarker [68,69].

Stavraka et al. [70] presented a novel strategy exploiting folate receptor alpha (FRα)as a target for cancer immunotherapy using the IgE antibody MOv18 IgE. Because of its high expression in many epithelial malignancies and its limited presence in normal tissues, FRα was used as a target antigen enabling the selective recognition of tumor cells. The authors demonstrated that MOv18 IgE retains its ability to bind FRα and activate immune mechanisms mediated by FcεRI and FcεRII receptors, thereby inducing immune-cell effector responses [70].The study also provided important information regarding the clinical safety of this strategy. Some patients developed localized non-allergic skin reactions associated with the activation of immune cells at the antibody administration site, but without features of a classical IgE-mediated anaphylactic reaction. These findings indicate that the use of IgE as an FRα-targeted therapeutic platform is clinically feasible and may represent an alternative to conventional IgG monoclonal antibodies. The MOv18 IgE strategy further supports FRα as a therapeutic target and a potential candidate biomarker for patient selection. However, whether quantitative FRα expression predicts clinical benefit from MOv18 IgE therapy remains to be established [70]. Despite promising clinical findings, FRα-targeted IgE immunotherapy remains at an early stage of development and requires a further evaluation of long-term safety, dose optimization, and the identification of patients most likely to derive therapeutic benefit.

The available evidence indicates that the clinical significance of FRα as a predictive biomarker is strongly dependent on the therapeutic modality, analytical method, and level of clinical validation. The most established application concerns membranous FRα protein expression assessed by a validated immunohistochemical companion diagnostic assay for selecting patients with platinum-resistant ovarian cancer for mirvetuximab soravtansine treatment. Clinical studies, particularly MIRASOL, support this treatment-selection strategy, although the restriction of enrollment to FRα-high populations limits conclusions regarding the magnitude of treatment benefit across the full spectrum of receptor expression [10,17,18,49].

In contrast, newer FRα-targeted antibody–drug conjugates are being developed with different molecular architectures, cytotoxic payloads, and mechanisms of intracellular drug release. Their potential activity in tumors with lower or heterogeneous FRα expression highlights the need for treatment-specific biomarker validation rather than the universal application of a single immunohistochemical cutoff. Furthermore, clinical efficacy observed within an FRα-defined subgroup should be distinguished from formal evidence that FRα expression modifies the relative benefit of treatment [50,51,52,53,54,64,65,66].

Other therapeutic approaches, including FRα-targeted CAR-T cells, radionuclide therapies, folate-functionalized nanocarriers, and receptor-directed immunotherapies, provide additional evidence of the biological relevance of FRα as a therapeutic target. However, much of the available evidence remains preclinical or derives from early clinical investigations, and standardized biomarker thresholds capable of predicting clinical benefit have not been established. Similarly, experimental associations between FOLR1 expression and resistance to conventional anticancer agents, including sorafenib, should not be interpreted as validated predictive relationships without prospective clinical confirmation [31,47,55,56,67,68,69,70].

Importantly, the different analytical modalities used to characterize FRα/FOLR1 are not interchangeable. FOLR1 mRNA abundance, membranous FRα protein expression, soluble FRα concentrations, and folate receptor-targeted imaging signals reflect different biological and methodological dimensions of receptor status. Their predictive relevance must therefore be evaluated within the specific therapeutic context in which they are intended to guide clinical decisions [8,9,10,17,33].

Overall, FRα represents a clinically actionable biomarker in a defined treatment setting and a promising molecular target for several emerging therapeutic strategies. Future studies should prioritize prospective biomarker-stratified designs, standardized analytical procedures, assessment of intratumoral heterogeneity, and formal evaluation of biomarker–treatment interactions. Such investigations will be essential to determine whether FRα expression can reliably predict therapeutic benefit beyond its currently established application in mirvetuximab soravtansine treatment [10,17,18,49,50,51,52,53,54].

## 6. FRα/FOLR1 as a Prognostic Biomarker

The significance of FRα as a potential prognostic marker has been investigated in numerous studies. Unlike its diagnostic value, which primarily concerns the presence or absence of the receptor in relation to the presence or absence of disease, the prognostic value of FRα relates to disease course, risk of recurrence, and patient survival and appears to depend on cancer type. Increased receptor expression in some malignancies has been associated with a more aggressive phenotype, although this relationship is not universal [71,72].

One of the most important studies summarizing the prognostic significance of FRα/FOLR1 is the meta-analysis by Liu et al. [71], which included 12 studies comprising 4471 patients with different types of cancer. The pooled analysis suggested an association between elevated FRα/FOLR1 expression and unfavorable overall survival (OS) and disease-free survival (DFS). Higher expression was also associated with adverse clinicopathological characteristics, including advanced disease stage, higher tumor grade, and lymph node involvement. However, the direction and magnitude of the prognostic association varied across cancer types. Therefore, these findings should not be interpreted as evidence that FRα represents a universal adverse prognostic biomarker. Rather, they highlight the need for cancer-specific analyses, standardized receptor assessment, and independent validation of prognostic associations [71].

Earlier studies, dating back to the previous century, had already provided evidence supporting a prognostic role for FRα. In some of these earlier studies, FRα was referred to as folate-binding protein (FBP); this historical terminology is retained below when describing the original findings. Toffoli et al. [73] assessed FRα expression in 99 patients with epithelial ovarian cancer treated with platinum-containing chemotherapy. FRα expression was measured in frozen ovarian tumor samples by flow cytometry using the MOv18 antibody. Among 58 patients with residual disease following primary surgery, high FBP expression was associated with an approximately 15-fold higher probability of failure to respond to chemotherapy. Importantly, FBP expression was not a significant predictor of survival in the overall study population; however, in the subgroup of patients with residual disease, high expression showed a trend toward poorer survival (HR 2.01; 95% CI 0.95–4.24). These findings suggest a potential association between FRα expression and chemotherapy response in a selected subgroup of patients with ovarian cancer. However, the absence of a statistically significant survival association in the overall cohort and the exploratory nature of the subgroup findings limit their prognostic interpretation. Moreover, the observed association with treatment response does not establish FRα as a predictive biomarker of platinum benefit without an appropriate comparative treatment analysis [73].

In another malignancy, breast cancer, Hartmann et al. [74] likewise demonstrated that strong FR expression was significantly associated with a poorer clinical course. The investigators analyzed folate receptor expression in 63 cases of invasive breast cancer. Among patients showing strong staining by immunohistochemistry (IHC), 17 out of 21 (81%) experienced disease recurrence, and median survival was 2.4 years. In comparison, recurrence occurred in 38% of patients with low expression, while median survival was not estimated. Importantly, the association between strong FR expression and adverse prognosis remained significant after adjustment for tumor size, lymph node involvement, estrogen receptor (ER) status, adjuvant treatment, histological type, and tumor grade (*p* < 0.001). These findings indicate that FR expression may provide prognostic information independently of conventional clinicopathological parameters [74].

A similar association was observed in patients with endometrial cancer. Brown Jones et al. [75] analyzed 310 tissue samples for the presence of FRα in the glandular epithelium of endometrial cancer cells. Moderate or strong FRα expression was detected in 41% of samples. High receptor expression was also significantly associated with adverse prognostic factors, including advanced disease stage, non-endometrioid histology, and high tumor grade. A significant association was also demonstrated between moderate/strong FRα expression and disease recurrence (*p* < 0.0014). The authors considered these findings supportive of both the potential use of FRα as a prognostic marker and the development of receptor-targeted therapies for high-risk endometrial cancers [75].

In a study by D’Angelica et al. [76] on colorectal cancer liver metastases, tissue from 160 patients who underwent resection of hepatic metastases was analyzed immunohistochemically for FRα expression. The favorable-outcome group comprised 56 patients who survived for at least 10 years after resection, whereas the poor-prognosis group consisted of 104 patients who died within 2 years after surgery. FRα was detected in 32% of patients in the early-death group compared with 13% of patients with longer survival (*p* = 0.03). Importantly, in multivariable analysis, FRα expression remained independently associated with outcome, together with the clinical risk score and surgical margin status. These findings indicate that FRα expression may also have prognostic value in advanced colorectal cancer with liver metastases [76].

Completely different results were obtained by Iwakiri et al. [72] in non-small-cell lung cancer (NSCLC). In adenocarcinoma, lower FOLR1 expression was associated, among other factors, with smoking, male sex, poorer histological differentiation, more advanced disease stage, higher T category in the TNM classification (T = size of the primary tumor), lymph node involvement, absence of an EGFR (epidermal growth factor receptor) mutation, and the presence of a TP53 mutation (TP53 encodes p53, which inhibits the division of damaged cells and initiates DNA repair or apoptosis). Importantly, patients with high FOLR1 expression had better 3-year overall survival (94.7% vs. 80.9%) and disease-free survival (75.4% vs. 60.8%). In multivariable analysis, high FOLR1 expression remained an independent favorable prognostic factor (*p* = 0.043). This study therefore represents an important example in which higher FOLR1 expression was associated with a more favorable rather than a poorer prognosis [72].

The findings concerning pancreatic cancer are particularly interesting because two independent studies reported opposing associations between FRα expression and survival. Cai et al. [77] analyzed FRα expression in 140 cases of pancreatic ductal adenocarcinoma (PDAC). Low, intermediate, and high expression was observed in 16%, 52%, and 32% of patients, respectively. Low FRα expression was associated with smoking, alcohol consumption, and lymphatic vessel involvement, as well as significantly shorter overall survival. Median OS was 15.1 months in the low-expression group, 24.0 months in the intermediate-expression group, and 36.3 months in the high-expression group. In multivariable analysis, FRα expression remained an independent prognostic factor (HR 0.61; *p* = 0.026), indicating that high FRα expression was associated with a more favorable prognosis in this population [77].

In contrast, Omote et al. [78] examined 100 patients with primary pancreatic cancer and obtained the opposite result. FRα expression was detected in 37% of cases. Patients with FRα-positive tumors had a shorter median overall survival than those with FRα-negative tumors (18.8 vs. 21.3 months), and FRα positivity was associated with an increased risk of death (HR 1.89; 95% CI 1.12–3.12; *p* = 0.017). In multivariable analysis, FRα, like the CA19-9 marker, remained an independent prognostic factor for OS. However, no significant difference in progression-free survival was observed between the groups [78].

The opposing findings reported by Cai et al. [77] and Omote et al. [78] demonstrate that the prognostic significance of FRα cannot be reduced to a universal relationship between increased receptor expression and unfavorable survival. Although both studies investigated pancreatic cancer, they reported associations in opposite directions. Such discrepancies may reflect differences in cohort characteristics, disease stage, immunohistochemical procedures, expression thresholds, statistical adjustment, and the biological heterogeneity of pancreatic tumors. Importantly, these findings also emphasize the need to distinguish statistically significant associations from reproducible, independently validated prognostic utility. Until these differences are resolved through standardized assessment and external validation, FRα should not be regarded as an established prognostic marker for routine clinical decision-making in pancreatic cancer [77,78].

In this context, the study by Tran et al. [79] provides complementary evidence because it was not a prognostic study but offered a mechanistic explanation for the potential variability of FRα expression in cancer cells. The authors demonstrated that glucocorticoid receptor activation can increase FRα expression in cancer cells. This indicates that FOLR1 levels may be regulated by transcription factors and the cellular environment. Thus, FRα expression may reflect not only the presence of a particular tumor phenotype, but also the biological state of the cell. From the perspective of prognostic assessment, this finding emphasizes the importance of considering the mechanisms regulating FOLR1 when interpreting differences in expression among malignancies [79].

Taken together, the available evidence indicates that the prognostic significance of FRα/FOLR1 is strongly dependent on cancer type and cannot be generalized across malignancies. Increased receptor expression has been associated with unfavorable outcomes in several tumor types, including breast and endometrial cancers and colorectal cancer liver metastases. Conversely, favorable prognostic associations have been reported in lung adenocarcinoma and in some pancreatic cancer cohorts, whereas other studies of pancreatic cancer have demonstrated the opposite relationship. These discrepancies highlight the importance of tumor-specific biological characteristics, analytical methodology, and patient population when interpreting prognostic associations [71,72,73,74,75,76,77,78].

Although several studies have identified FRα/FOLR1 expression as an independent factor in multivariable survival analyses, statistical independence within an individual cohort does not necessarily establish clinical prognostic utility. External validation, standardized expression thresholds, reproducible analytical procedures, and a demonstration of incremental prognostic information beyond established clinicopathological factors remain necessary before routine implementation can be justified [71,72,74,76,77,78].

Furthermore, prognostic and predictive evidence must be distinguished. Associations between FRα/FOLR1 expression and overall survival, disease-free survival, or recurrence primarily inform prognosis, whereas relationships between receptor status and benefit from a specific therapy require treatment-focused evaluation. In particular, an association with response to chemotherapy does not establish a predictive biomarker unless the influence of receptor status on treatment benefit is adequately investigated. Therefore, FRα/FOLR1 should currently be considered as a potentially informative, cancer-type-dependent prognostic biomarker rather than a universally validated indicator of clinical outcome [71,72,73,77,78].

## 7. Conclusions

The clinical utility of FRα/FOLR1 varies substantially across cancer types and depends on the biological analyte, assessment method, and intended clinical application. To distinguish established clinical applications from emerging and investigational uses, Table 3 summarizes the available evidence regarding the diagnostic, prognostic, and predictive relevance of FRα/FOLR1 across malignancies, together with the corresponding assessment methods and levels of clinical evidence.

The strongest evidence supporting the clinical utility of FRα concerns its role in treatment selection for patients with FRα-high, platinum-resistant ovarian, fallopian tube, or primary peritoneal cancer. Membranous FRα expression assessed using a validated immunohistochemical companion diagnostic assay identifies patients eligible for mirvetuximab soravtansine, for whom randomized phase III evidence has demonstrated improved clinical outcomes compared with chemotherapy. Nevertheless, the clinical applicability of the validated expression threshold is specific to this therapeutic context and should not be extrapolated to other FRα-directed agents. Furthermore, the available randomized evidence does not fully establish how relative treatment benefit varies across the entire spectrum of FRα expression [10,17,18,49].

In contrast, the broader diagnostic and prognostic applications of FRα/FOLR1 remain investigational. Circulating soluble FRα and folate receptor-targeted molecular imaging have shown promising results in selected malignancies, but their clinical implementation is limited by insufficient external validation, the absence of standardized thresholds, and uncertainty regarding their incremental value over established diagnostic approaches. Similarly, the prognostic significance of FRα/FOLR1 is cancer-dependent, with favorable, unfavorable, and conflicting associations reported across different tumor types. These findings underscore the importance of distinguishing FOLR1 mRNA expression, membranous FRα protein, circulating soluble FRα, and receptor-targeted imaging signals, which represent biologically and analytically distinct measurements [8,9,25,34,35,36,45,46,71,72,73,74,75,76,77,78].

The growing pipeline of FRα-directed therapies, including novel antibody–drug conjugates, cellular immunotherapies, folate-based conjugates, and radionuclide approaches, further highlights the therapeutic relevance of this receptor. However, evidence of target expression or receptor-dependent drug delivery does not, by itself, establish predictive biomarker utility. Establishing their predictive utility will require treatment-specific biomarker assessment strategies, reproducible assays, and prospective evidence linking defined biomarker measurements to therapeutic benefit. Particular attention should also be given to intratumoral heterogeneity, receptor accessibility, off-target uptake, and treatment-related toxicity [31,47,50,51,52,53,54,55,56,61,62,63,64,65,66].

Overall, FRα/FOLR1 should be regarded as a clinically actionable biomarker within a clearly defined therapeutic setting, while its broader diagnostic, prognostic, and predictive applications require further validation. Future investigations should prioritize analytical standardization, external validation, and prospective studies demonstrating that FRα/FOLR1 assessment improves clinical decision-making beyond existing approaches.

## Figures and Tables

**Table 1:** Selected folate receptor-targeted radiotracers investigated for oncological imaging: experimental models and clinical evidence.

Folate-Based Radiotracer	Radionuclide	FR Targeting/Selectivity	Imaging Technique	Cancer Type/Investigated Model	Study Design/Evidence Type	Reference
^111^In-DTPA-folate	^111^In	FR-targeted; FRα/FRβ selectivity not demonstrated	SPECT	Ovarian cancer	Initial clinical imaging study	[34]
^99m^Tc-EC20 (etarfolatide)	^99m^Tc	FR-targeted; FRα/FRβ selectivity not demonstrated	SPECT	FR-positive solid tumors; predominantly renal cell carcinoma	Exploratory clinical imaging study (154 patients)	[35]
^18^F-AzaFol (3′-aza-2′-[^18^F] fluorofolic acid)	^18^F	FR-targeted; no preferential FRα selectivity demonstrated in comparative preclinical assays	PET	Lung adenocarcinoma	Prospective, multicenter, first-in-human dosimetry study (6 patients)	[36]
^18^F-6*R*-aza-5-methyltetrahydrofolate	^18^F	Preferential FRα selectivity demonstrated experimentally	PET/CT	FRα-positive RT16 and FRβ-positive D4 xenografts	Preclinical in vitro binding studies, biodistribution and in vivo PET/CT	[25]
^68^Ga-NOTA-folate	^68^Ga	FR-targeted; FRα/FRβ selectivity not established	PET/CT	FR-positive KB xenografts; KB and A549 cell lines used in vitro	Preclinical in vitro and in vivo imaging study	[37]
^44^Sc-DOTA-folate	^44^Sc	FR-targeted; FRα/FRβ selectivity not established	PET	FR-positive KB tumor models	Preclinical in vitro and in vivo evaluation	[38]
^152^Tb-DOTA-folate	^152^Tb	FR-targeted; FRα/FRβ selectivity not established	PET	FR-positive KB tumor xenografts	Preclinical proof-of-concept imaging study	[39]
Folate-NOTA-Al^18^F	^18^F	FR-targeted; FRα/FRβ selectivity not established	PET	FR-positive tumor models	Preclinical radiotracer synthesis and imaging evaluation	[40]

Note: FR, folate receptor; FRα, folate receptor alpha; FRβ, folate receptor beta; 5-MTHF, 5-methyltetrahydrofolate; PET, positron emission tomography; SPECT, single-photon emission computed tomography; CT, computed tomography. The reported cancer types and experimental models correspond to the cited primary studies. FR-targeted radiotracers should not be considered FRα-selective unless preferential binding to FRα over FRβ has been experimentally demonstrated. The absence of demonstrated subtype selectivity does not establish equivalent binding to both receptor isoforms. Preclinical receptor-targeted imaging findings do not, by themselves, establish clinical diagnostic or predictive utility.

**Table 2:** FRα-targeted antibody–drug conjugates: mechanisms of action, potential clinical applications, and development status.

FRα-Targeted ADC	ADC Construct and Cytotoxic Payload	Mechanism of Action	Potential Clinical Application	Regulatory/Development Status	Reference
Mirvetuximb soravtansine (MIRV, IMGN853, Elahere^®^)	Anti-FRα IgG1 monoclonal antibody M9346A conjugated through the disulfide-containing sulfo-SPDB linker to the maytansinoid DM4;	FRα binding and receptor-mediated internalization → lysosomal degradation of the antibody → linker-dependent intracellular processing and formation of DM4-containing metabolites → inhibition of tubulin polymerization, mitotic arrest and cell death; membrane-permeable metabolites contribute to a bystander effect	FRα-positive, platinum-resistant epithelial ovarian, fallopian tube, or primary peritoneal cancer; patient selection based on the validated VENTANA FOLR1 (FOLR1-2.1) RxDx IHC assay (≥75% of viable tumor cells showing moderate-to-strong membranous staining)	FDA accelerated approval 2022; full approval 2024	[18,49]
Farletuzumab ecteribulin (MORAb-202)	Humanized anti-FRα antibody farletuzumab conjugated through a cathepsin-B-cleavable linker to the microtubule inhibitor eribulin	FRα binding → internalization → lysosomal linker cleavage → release of eribulin → inhibition of microtubule dynamics and tumor-cell death; bystander activity has also been demonstrated	Investigational therapy for FRα-expressing solid tumors, particularly ovarian cancer; also evaluated in other FRα-expressing malignancies	Clinical development; Phase I clinical data available	[62,63]
Luveltamab tazevibulin (STRO-002; luvelta)	Site-specific anti-FRα ADC containing a cleavable 3-aminophenyl hemiasterlin linker-warhead (SC239); intracellular release of the tubulin-targeting 3-aminophenyl hemiasterlin SC209	Rapid FRα-dependent internalization → intracellular release of SC209 → disruption of microtubule dynamics and tumor-cell death; bystander killing has been demonstrated	Previously investigated in FRα-expressing ovarian, endometrial and other cancers	Clinical development terminated following portfolio reprioritization	[64]
Rinatab artsesutecan (Rina-S; GEN1184; formerly PRO1184)	FRα-directed ADC incorporating a highly hydrophilic, protease-cleavable linker and the topoisomerase I inhibitor exatecan	FRα-mediated internalization → proteolytic linker cleavage and intracellular exatecan release → TOP1 inhibition, DNA damage and tumor-cell death	Ovarian and other FRα-expressing solid tumors; clinical development also includes populations irrespective of FRα expression level	Phase III clinical development	[65,66]
Torvutatug samrotecan (AZD5335)	FRα-targeted ADC incorporating a topoisomerase I inhibitor payload	FRα-dependent internalization → intracellular TOP1 inhibitor release → DNA damage response and tumor-cell death; antitumor activity has been demonstrated across models with high and low FRα expression	Ovarian and other FRα-expressing cancers, including tumors with lower FRα expression	Phase III–TREVI-OC-01	[50]
Sofetabart mipitecan (LY4170156; formerly MBK-103)	Humanized anti-FRα IgG1 antibody with silenced Fc function, proprietary polysarcosine hydrophobicity-masking moiety, dipeptide-cleavable linker and exatecan payload	FRα-dependent internalization → intracellular release of exatecan → TOP1 inhibition and DNA damage; preclinical activity has been observed across different levels of FRα expression	Platinum-resistant and platinum-sensitive ovarian cancer and other FRα-expressing solid tumors	Phase III–FRAmework-01	[51,52]
IMGN151	Asymmetric, bivalent, biparatopic anti-FRα antibody targeting two independent FRα epitopes, conjugated through a stable cleavable peptide linker to the maytansinoid DM21	Biparatopic FRα binding → receptor-mediated internalization → intracellular DM21 release → disruption of microtubule dynamics and tumor-cell death; designed to retain activity across heterogeneous FRα expression	Investigational therapy for ovarian, endometrial and other gynecologic cancers, including tumors with heterogeneous FRα expression	Phase I clinical development	[53,54]

Note: ADC, antibody–drug conjugate; FRα, folate receptor alpha; TOP1, topoisomerase I. Mirvetuximab soravtansine is an approved FRα-targeted ADC with an established IHC-based patient-selection strategy in a defined clinical setting. The other agents listed are investigational or have undergone discontinuation of clinical development; their inclusion does not imply that FRα expression has been clinically validated as a predictive biomarker for each agent.

**Table 3:** Diagnostic, prognostic, and predictive utility of FRα/FOLR1 across malignancies: Clinical evidence and limitations.

Cancer/Setting	Clinical Role	Analyte/Method	Principal Finding	Evidence and Clinical Interpretation	Reference
Platinum-resistant ovarian, fallopian tube, or primary peritoneal cancer	Predictive/treatment selection (established clinical application)	Membranous FRα IHC; VENTANA FOLR1 RxDx (≥75% tumor cells with 2+/3+ staining)	MIRASOL showed improved ORR, PFS and OS with mirvetuximab soravtansine versus chemotherapy in FRα-high disease	Randomized phase III; established for this specific indication. Threshold and benefit cannot be extrapolated to other agents or FRα-low tumors.	[10,17,18]
Ovarian cancer	Diagnostic (exploratory)	Serum soluble FRα; study-specific assays	Promising diagnostic discrimination, including favorable results versus CA125 in a prospective cohort (n = 231)	Observational; no independently validated threshold or established screening/standalone diagnostic role.	[9,26]
Prostate cancer	Diagnostic (exploratory)	Serum FRα by ELISA	Case–control study (45 cases; 45 controls): AUC 0.770	Small observational study; insufficient for routine standalone diagnosis.	[45]
Hepatocellular carcinoma	Diagnostic and prognostic (exploratory)	Serum FRα protein; tumor FOLR1 transcript assessed separately	Serum FRα complemented AFP in diagnostic analyses and was associated with overall survival	Observational cohort; requires external validation and clinical cutoffs. Serum protein and tumor mRNA are distinct analytes.	[46]
Selected FR-positive solid tumors	Molecular imaging (exploratory)	Folate-targeted SPECT/PET (^111^In-DTPA-folate, ^99m^Tc-EC20, ^18^F-AzaFol)	Early human studies established feasibility, tracer distribution and/or dosimetry	Early-phase feasibility, not validated diagnostic performance. Signal may also reflect FRβ-positive immune cells	[34,35,36]
Breast, endometrial and colorectal liver metastases	Prognostic (exploratory)	Tumor FRα/receptor staining; study-specific tissue assays	Adverse outcome associations reported in breast (n = 63), endometrial (310 evaluable) and selected resected colorectal liver-metastasis cohorts	Retrospective observational evidence; heterogeneous populations, assays and sampling; no validated routine prognostic cutoff.	[74,75,76]
Non-small-cell lung cancer	Prognostic (exploratory)	Tumor FOLR1 mRNA expression (not FRα IHC)	Higher FOLR1 transcript levels were associated with more favorable outcomes in resected NSCLC, particularly adenocarcinoma.	Retrospective observational evidence; gene expression must not be equated with membranous FRα protein.	[72]
Pancreatic ductal adenocarcinoma	Prognostic (inconsistent)	Tumor FRα immunostaining	Two observational cohorts reported opposing directions of survival association	Conflicting findings preclude clinical prognostic use without assay standardization and external validation.	[77,78]
Ovarian cancer treated with platinum	Treatment-response association (exploratory)	Tumor folate-binding protein/FRα by flow cytometry	Historical subgroup association with chemotherapy response among patients with residual disease	Observational, not a validated predictive biomarker; no treatment-by-biomarker interaction established	[73]
FRα-directed investigational agents	Therapeutic targeting (investigational)	Drug-specific FRα assays/eligibility criteria	New ADCs, CAR-T and folate–drug conjugates have heterogeneous preclinical, early clinical and trial-design evidence	Do not conflate published phase I findings, preclinical data, conference abstracts or trial registrations with completed phase III efficacy or validated selection thresholds	[50,51,52,53,54,55,56,61,62,63,64,65,66]

Note: ADC, antibody–drug conjugate; AFP, alpha-fetoprotein; AUC, area under the curve; FRα/FRβ, folate receptors alpha/beta; IHC, immunohistochemistry; ORR, objective response rate; OS, overall survival; PFS, progression-free survival; SPECT/PET, single-photon emission computed tomography/positron emission tomography. Evidence categories are descriptive, not formal GRADE ratings. The table distinguishes FOLR1 gene expression, tumor FRα protein, circulating FRα and receptor-targeted imaging; these measures are not interchangeable.

## Data Availability

No new data were created or analyzed in this study. Data sharing is not applicable to this article.
